# Diseases of Nose and Throat

**Published:** 1895-01

**Authors:** 


DISEASES OF THE NOSE AND THROAT. By Watson Williams, M. D., London. Colored plates. $2.75.

